# Trends in the incidence of primary liver and biliary tract cancers in England and Wales 1971–2001

**DOI:** 10.1038/sj.bjc.6603127

**Published:** 2006-05-30

**Authors:** J West, H Wood, R F A Logan, M Quinn, G P Aithal

**Affiliations:** 1 Division of Epidemiology and Public Health, University of Nottingham Medical School, Queen's Medical Centre University Hospital, Nottingham NG7 2UH, UK; 2 National Cancer Intelligence Centre, Office for National Statistics, London, UK; 3 Wolfson Digestive Diseases Centre, Queen's Medical Centre University Hospital, D Floor, South Block, Nottingham NG7 2UH, UK

**Keywords:** epidemiology, liver disease, biliary disease, incidence

## Abstract

In the last two decades, mortality from primary liver cancer has increased in the UK. We aimed to determine whether the incidence trends for these cancers were similar and in particular if the increasing occurrence of cholangiocarcinoma has continued. We calculated directly age-standardised incidence rates (using the European standard population) by subsite and histological type for all cancers of the liver, gallbladder and biliary tract in England and Wales from 1971 to 2001, using cancer registry data. The incidence of cancers of the liver, gallbladder and biliary tract increased, with the greatest rise, around 12-fold, in intrahepatic bile duct cancers. The rate of liver cell cancer increased by around 45% in males, but by <10% in females. There were marked reductions in the incidence of gallbladder and extrahepatic bile duct cancer. Cholangiocarcinoma increased around 16-fold and became the most common type of primary liver cancer in females, while hepatocellular carcinoma remained the commonest type in males. The age-specific incidence rates showed that intrahepatic bile duct cancer continued to increase throughout the 1990s in those aged 75 and over, while liver cell cancer decreased in the older age groups. In conclusion, there were increases in the incidence of primary liver cancer, which have been particularly dramatic for intrahepatic bile duct cancer, over the last three decades of the 20th century in England and Wales. There has been a halving in the incidence of gallbladder cancer and a reduction of a third in extrahepatic bile duct cancer.

Studies from the United States of America (USA) and the United Kingdom (UK) have shown marked increases in mortality from primary liver cancers in the last two decades of the 20th century (Taylor-Robinson *et al*, 1997; El-Serag and Mason, 1999; Patel, 2001; Taylor-Robinson *et al*, 2001; Shaib *et al*, 2004). While there have been incidence data reported from the USA, there has been no report of recent trends (last 30 years) in incidence from the UK. The data from the USA have shown that the incidence of, as well as mortality from, both hepatocellular carcinoma and intrahepatic cholangiocarcinoma has risen dramatically (El-Serag and Mason, 1999; Patel, 2001). The incidence rates of intra- and extrahepatic cholangiocarcinoma in the USA, however, show divergent trends, which may reflect changes in the classification of these malignancies resulting from better diagnostic and histological techniques. Nevertheless, it has been argued that the increase in the incidence of intrahepatic cholangiocarcinoma is real, as there has been no increase in the proportion of patients with either more localised disease or a decrease in tumour size at diagnosis (Shaib *et al*, 2004). However, reclassification of cases coded as carcinoma not otherwise specified (NOS) or extrahepatic cholangiocarcinoma, which that study was unable to explore (Shaib *et al*, 2004), could have contributed to the rise.

Since survival from cancers of the liver and gallbladder is quite low, trends in mortality should be a reasonable proxy for trends in incidence. However, a higher proportion of deaths than cases are coded to ‘site unspecified’, indicating that diagnostic accuracy is less certain for mortality than incidence. In addition, cancer registrations include information about the histological classification of tumours, which is not available from a death certificate. By using incidence data and examining the trends in incidence by subsite and histology, we will be able to more accurately describe the trends in cancers of the liver and biliary tract, and to explore whether there are any important differences between incidence and mortality trends. We will also be able to see if the rise in cholangiocarcinoma and reduction in extrahepatic biliary and gallbladder cancers has continued into the 21st century.

## MATERIALS AND METHODS

We used data collected in England by the nine regional cancer registries, and in Wales by the Welsh Cancer Intelligence and Surveillance Unit. A subset of the collected data for each patient (the minimum data set) is sent to the National Cancer Intelligence Centre at the Office for National Statistics (ONS). The data are loaded onto a person-based database and validated (Office for National Statistics, 2004). Validation includes checking the compatibility of the cancer site and the associated histology.

We used validated registrations of cancers diagnosed from 1971 to 2001 for our analyses. The site codes of the International Classification of Diseases used to identify and group cases of liver, gallbladder and biliary tract cancer are shown in Table 1a. International Classification of Disease (ICD8) (1967) was used for cases diagnosed from 1971 to 1978, International Classification of Disease (ICD9) (1975) from 1979 to 1994, and International Classification of Disease (ICD10) (1992) from 1995 onwards. Cases were excluded from the analysis if the behaviour of the tumour was classified as benign, uncertain or *in situ* (<0.5% of the cases registered from 1971 to 2001), leaving malignant primary tumours, and a small number of malignant secondary tumours and tumours not defined as primary or secondary (<4% of cases).

In addition to ICD site code, cases of cancer were classified by histology code: those diagnosed from 1971 to 1978 using the Manual of Tumor Nomenclature and Coding (MOTNAC) (1968), those from 1979 to 1994 using the International Classification of Disease for Oncology (ICD-O) (1976) and those from 1995 onwards using the second edition of this volume (International Classification of Disease (ICD-O2), 1990). Cases were grouped according to the following categories: neoplasm not otherwise specified (NOS), code M800; carcinoma NOS, M801; adenocarcinoma, M814; hepatocellular carcinoma, M817; cholangiocarcinoma, M816; and ‘other’ (all other codes) (see Table 1b). To assess whether the proportion of registered cases that were diagnosed on the basis of histological verification from a biopsy has changed over time, we extracted data from 1993 to 2001. This information was only routinely recorded on cancer registrations from 1993 onwards.

We calculated age-specific incidence rates for males and females, and directly age-standardised rates were calculated by weighting the age-specific rates according to the European standard population. Rolling averages for 3 years were applied to age-standardised rates, and 5-year rolling averages were applied to age-specific rates, to smooth the trends over time. We calculated rate ratios and 95% confidence intervals (CI) to compare the rates between the 3-year periods at the beginning and end of the study period.

All statistical analysis was carried out using Excel software. Statistical significance was taken as *P*<0.05.

## RESULTS

**Figure 1:**
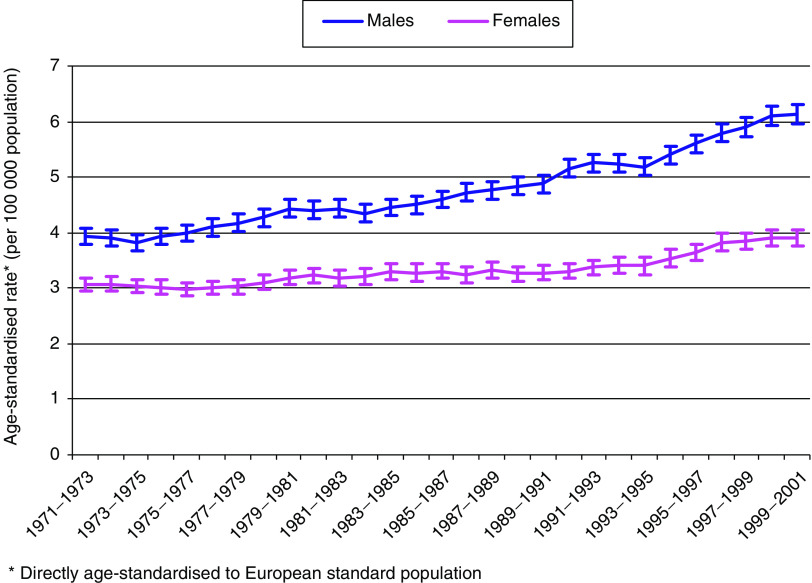
Trends in age-standardised incidence rates from 1971 to 2001 in England and Wales of all malignant cancers of the liver, gallbladder and biliary tract, by sex (3-year rolling averages). Bars are standard errors.

In 1999–2001, the overall age-standardised rate of cancers of the liver, gallbladder and biliary tract was around 60% higher in males than in females (6.13 and 3.89 per 100 000 population, respectively; Table 2 and Figure 1). By comparison, in 1971–73, the rate was only about 30% higher in males (3.93 and 3.06 per 100 000, respectively). The incidence rates for cancers of the liver, gallbladder and biliary tract increased by 56% (95% CI 46–66%) in males and 27% (95% CI 18–37%) in females between 1971–73 and 1999–2001.

### Incidence by subsite

**Figure 2:**
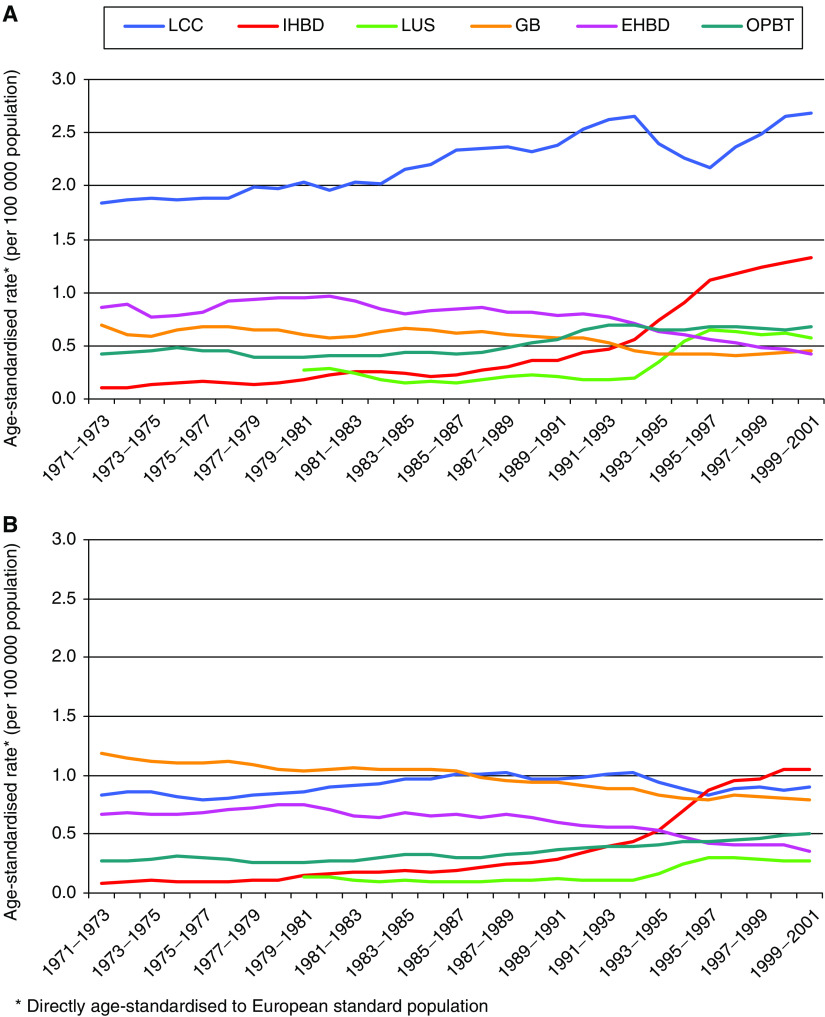
Trends in age-standardised incidence rates of all malignant cancers of the liver, gallbladder and biliary tract, by subsite from 1971 to 2001 in England and Wales for (**A**) males and (**B**) females (3-year rolling averages). Liver cell cancer (LCC); cancer of the intrahepatic bile ducts (IHBD); cancer of the liver, unspecified (LUS); cancer of the gallbladder (GB); cancer of the extrahepatic bile ducts (EHBD); and cancer of other parts of the biliary tract (OPBT). For ICD site codes, see Table 1a.

In 1971–73, cancer of the intrahepatic bile ducts was rare in both males and females (age-standardised rates 0.11 and 0.09 per 100 000, respectively), but over the subsequent three decades the incidence rates increased greatly, and were around 12 times higher at the end of the study period (1.33 and 1.06 per 100 000, respectively; Table 2 and Figure 2). The rate of liver cell cancer increased significantly by 46% (95% CI 31–60%) in males over the period, from 1.84 to 2.68 per 100 000, while in females there was only a small, nonsignificant increase (from 0.84 to 0.91 per 100 000; 8% increase 95% CI −9 to +26%).

In both males and females, the rate of cancers of the liver unspecified doubled and those of other parts of the biliary tree increased by about 60–90% over the study period. There were significant decreases in the rates of cancer of the gallbladder (around 30%) and also of cancer of the extrahepatic bile ducts (around 50%) between 1971–73 and 1999–2001 in both sexes.

In 1971–73, liver cell cancer was the most common subsite in males and this remained the case in 1999–2001; the incidence rate was around twice as high as that of cancer of the intrahepatic bile ducts (2.68 and 1.33 per 100 000, respectively). In females, however, the rate of cancer of the intrahepatic bile ducts increased by such a large amount over the study period that by 1999–2001 the rates of liver cell cancer and intrahepatic bile duct cancer were similar (0.91 and 1.06 per 100 000, respectively). In 1971–73, the most common subsite in females was the gallbladder, but in 1999–2001 intrahepatic bile duct was the most common, closely followed by liver cell cancer.

It should be noted that, with the change for the coding of cancers from ICD9 to ICD10 in 1995, which resulted in changes in published rates (Rooney and Devis, 1996; Brock *et al*, 2004), there were decreases in the rates of liver cell cancer (by just under 30% between 1994 and 1995) and corresponding increases in the rates of cancer of the intrahepatic bile ducts (by 60–70% between 1994 and 1995) in both sexes. In addition, the rates of cancers of the liver unspecified increased (by about twofold between 1994 and 1995). These changes did not in general affect the underlying trends in the rates of these types of cancer over the study period.

### Age-specific incidence by subsite

**Figure 3:**
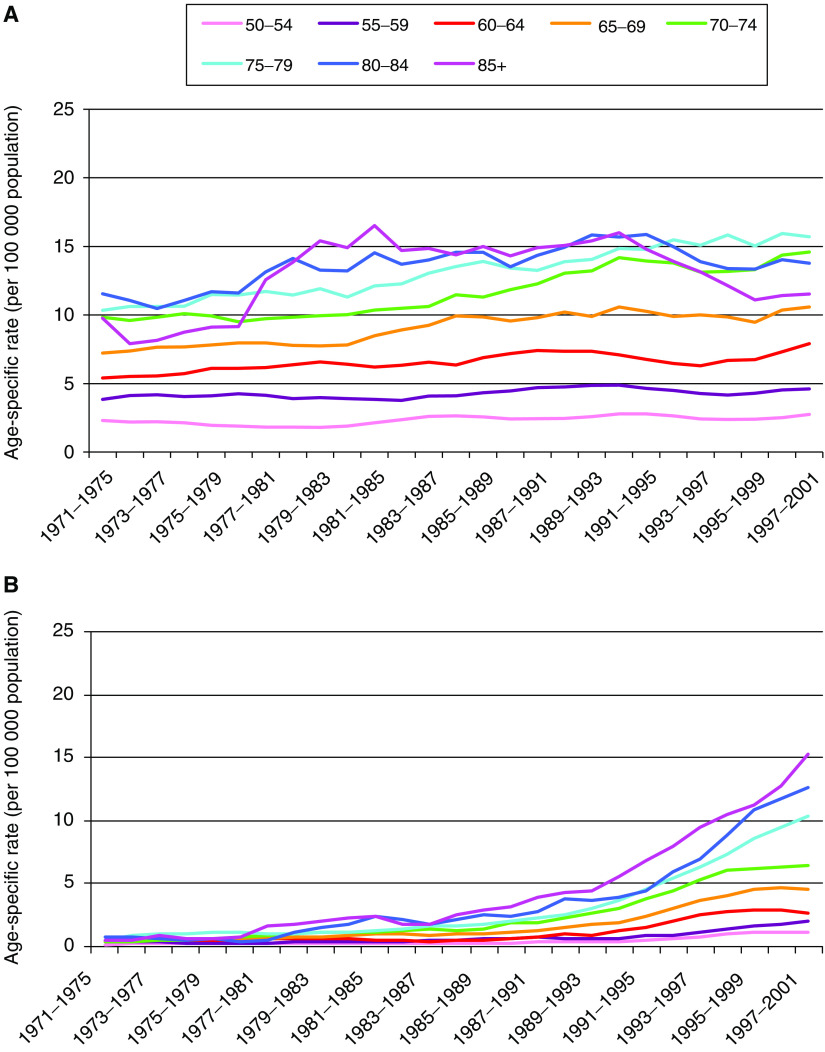

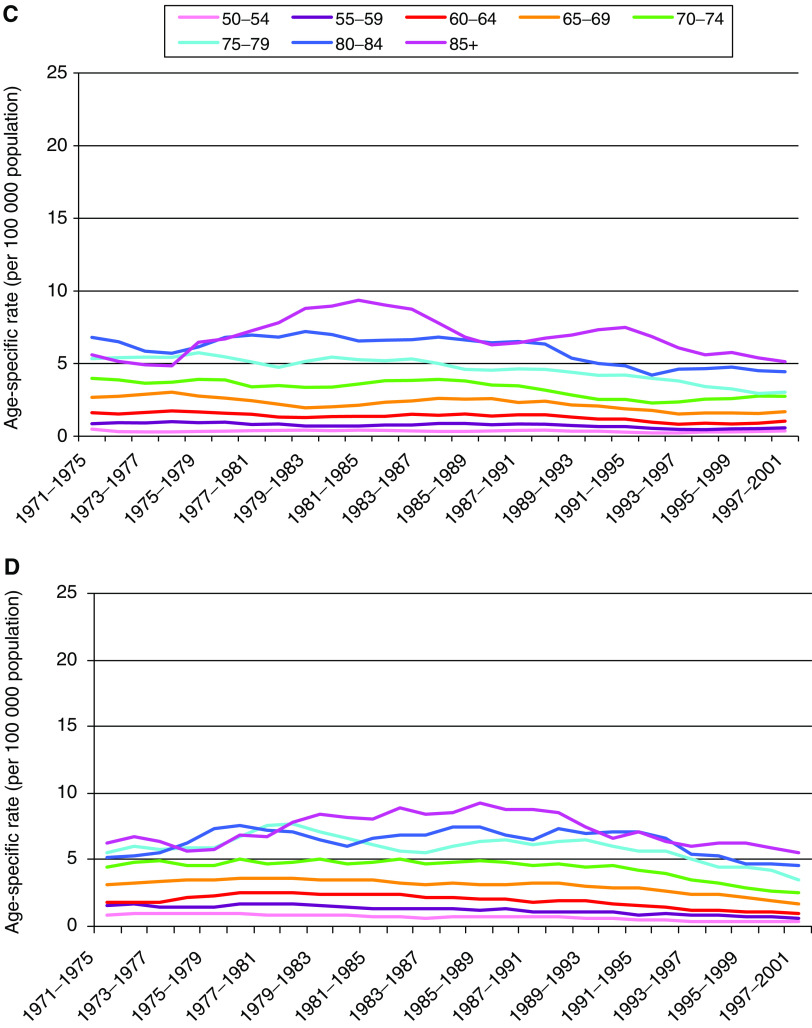
Trends in age-specific incidence rates in males from 1971 to 2001 in England and Wales of (**A**) liver cell cancer, (**B**) cancer of the intrahepatic bile ducts, (**C**) cancer of the gallbladder and (**D**) cancer of the extrahepatic bile ducts (3-year rolling averages).

The age-specific incidence rates in males for liver cell cancer and cancer of the intrahepatic bile ducts are shown in Figure 3A and B, respectively. The two figures show clear differences between the two subsites, with a gradual increase in the rates of liver cell cancer across the study period for all except the oldest age groups, where the rates rose in the 1970s and fell in the 1990s. In contrast, the rates of intrahepatic bile duct cancer increased quite slowly in all age groups up to about 1990, and this was followed by a rapid increase, which then levelled off in most of the age groups under 75 in the mid- to late 1990s.

The incidence of gallbladder and extrahepatic bile duct cancers decreased markedly across all age groups from the late 1980s onwards (Figure 3c and d, respectively). The trends in females were similar (data not shown).

### Incidence by histology

**Figure 4:**
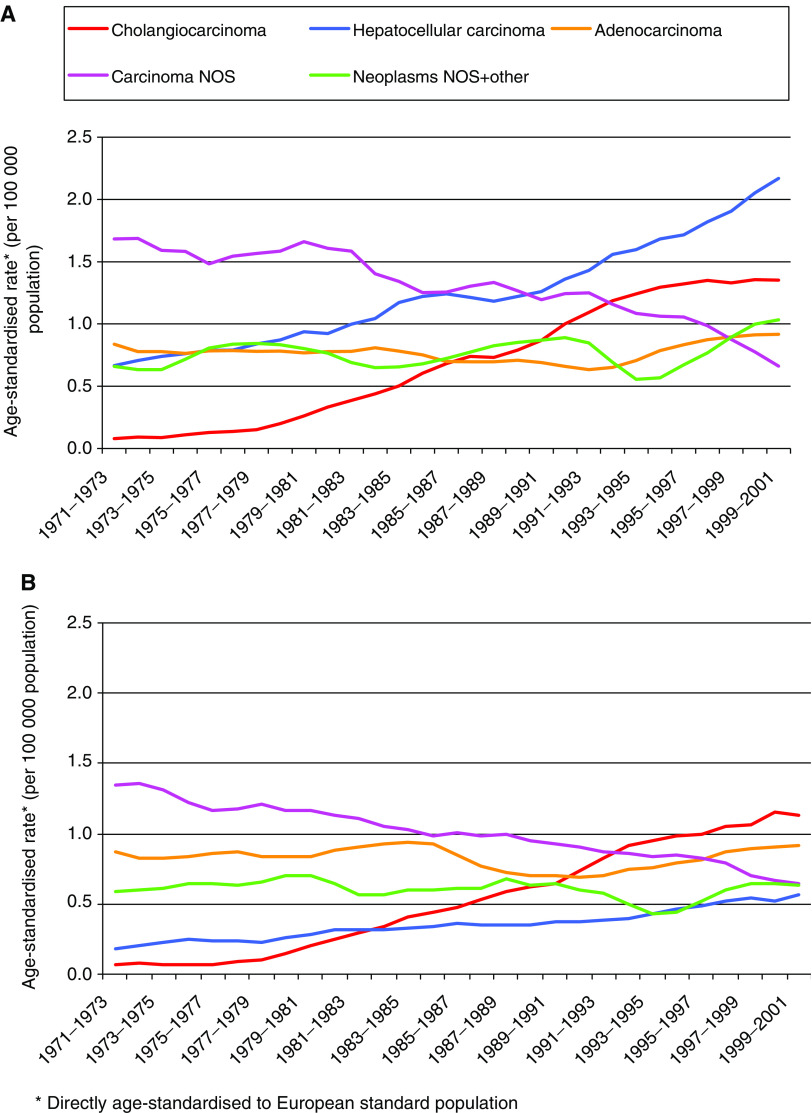
Trends in age-standardised incidence rates of all malignant cancers of the liver, gallbladder and biliary tract by histological type from 1971 to 2001 in England and Wales for (**A**) males and (**B**) females (3-year rolling averages). For histology codes, see Table 1b.

In 1971–73, the incidence rates of cholangiocarcinoma were very low and were similar in males and females (0.08 and 0.07 per 100 000, respectively). Incidence increased markedly and was around 16 times higher at the end of the study period than at the beginning in both males and females (1.35 and 1.13 per 100 000, respectively, in 1999–2001; Figure 4). Although it was the least common histological type in 1971–73, cholangiocarcinoma was the most common in females in 1999–2001, followed by adenocarcinoma. The incidence of hepatocellular carcinoma was around three times higher at the end of the study period than at the beginning in both males and females, but was close to four times higher in males than in females throughout. Hepatocellular carcinoma was the least common histological type in females in 1999–2001 (13% of all cancers of the liver, gallbladder and biliary tract), but in males it had become the most common type (34% of all cancers of the liver, gallbladder and biliary tract).

The incidence rates of adenocarcinoma, carcinoma NOS and neoplasm NOS and any other type not specified (neoplasm NOS+other) were similar in males and females in 1971–73 and followed similar trends across the following three decades. The rates of adenocarcinoma in both sexes and neoplasm NOS+other in females did not change significantly, while those of carcinoma NOS decreased by 50% in females and 60% in males, and those of neoplasm NOS+other increased by over 50% in males over the study period.

The proportion of cancers of the liver, gallbladder and biliary tract that were diagnosed on the basis of histological verification from a biopsy increased from 41% in 1993 to 48% in 2001. Of the cases that were not histologically verified, the majority were clinically diagnosed (58% on average in the period 1993–2001).

### Histology by subsite

Between 1971 and 2001, there was a large increase in the proportion of intrahepatic bile duct cancers coded as cholangiocarcinoma, and corresponding decreases in the proportions coded as adenocarcinoma and carcinoma NOS, indicating a move towards more specific coding of histological types. A similar pattern was observed for cancers of the extrahepatic bile ducts and for liver cell cancers.

In 2001, hepatocellular carcinoma was used to classify only liver cell cancers or cancers of the liver unspecified (with the exception of a very small number of cases in other subsites). In all, <10% of cancers of the liver unspecified were hepatocellular carcinomas, with the majority classified as carcinoma NOS or neoplasm NOS+other (Table 3). The proportion of liver cell cancers classified as hepatocellular carcinoma differed between the sexes – over 80% in males and just under 60% in females. This difference was mainly due to a higher proportion being coded as carcinoma NOS in females (19 *vs* 3% in males).

In both sexes, around 80% of intrahepatic bile duct cancers were coded as cholangiocarcinoma, compared to around 30–40% of extrahepatic bile duct cancers. For cancers of the liver unspecified, gallbladder and other parts of the biliary tract, cholangiocarcinoma accounted for <10% of cases (Table 3).

## DISCUSSION

In England and Wales, the incidence of cancers of the liver, gallbladder and biliary tract increased over the last three decades of the 20th century, particularly in males. The rates of intrahepatic bile duct cancer increased dramatically in both sexes, whereas the rate of liver cell cancer increased significantly in males, but not in females. In 1999–2001, liver cell cancer was the most common subsite in males and was twice as common as intrahepatic bile duct cancer. In contrast, intrahepatic bile duct cancer was the most common subsite in females. These incidence trends show some divergence from the previously reported mortality statistics. In addition, there were dramatic reductions in the incidence of extrahepatic bile duct and gallbladder cancers in both men and women.

In terms of histological type, the trends were quite similar between the sexes. The incidence of cholangiocarcinoma increased by around 16-fold, and that of hepatocellular carcinoma by threefold over the last three decades of the 20th century. These increases were too large to be accounted for by a shift in coding from other histological types over the period. Our examination of the agreement between subsite and histological type showed that over 80% of cholangiocarcinomas were intrahepatic (coded as intrahepatic bile duct cancer) and around 95% of hepatocellular carcinomas were coded as liver cell cancer.

Our use of cancer incidence data has provided the opportunity to examine long-term incidence trends for these cancers in England and Wales, where only mortality data have recently been reported. The information on histological type of each cancer, the proportion diagnosed on the basis of histology and our age-specific analyses have allowed greater examination of the possibility of ascertainment bias than is possible with mortality statistics. As might be expected, the trends in incidence are similar to those reported for mortality in England and Wales. Indeed, the mortality trends in both liver cell cancer and intrahepatic bile duct cancer for both men and women up until 1996 showed, as we do, that in women intrahepatic bile duct cancer was the most common type, in contrast to men, in whom liver cell cancer was most common. It might be argued that the increase we show in intrahepatic bile duct cancer reflects greater ascertainment consequent upon improved endoscopic and radiological techniques. Indeed, this interpretation has some support, since we found that the proportion of all liver, gallbladder and biliary tract cancers that were diagnosed on the basis of histological verification from a biopsy increased between 1993 and 2001. It is also possible that some of the increase in intrahepatic bile duct cancer may be due to diagnostic transfer from cases that were previously coded as extrahepatic bile duct cancer, although the reductions in extrahepatic bile duct cancer over the period were small.

Imaging modalities such as computerised tomography (CT) and endoscopic retrograde cholangiopancreatography (ERCP) have been available in most UK hospitals since the mid-1980s (McCune, 1988; Scott and Atkinson, 1989), but the increase in the incidence of intrahepatic bile duct cancer has continued at the same rate even in the last decade. Therefore, while some of the rise might be explained by increased ascertainment (i.e. increased use of imaging modalities over time in line with the increasing incidence), and certainly the increase in the proportion of cases diagnosed on the basis of histology suggests that this is partly responsible, we believe that this is not sufficient explanation. In particular, our analysis by histological type showed that the increase in the incidence of cholangiocarcinoma was far greater than the decreases in carcinoma NOS and neoplasm NOS+other. The marked decrease in gallbladder cancer is also unlikely to be anything other than a true trend, as ascertainment is unlikely to be an issue for this particular malignancy. Furthermore, diagnostic transfer does not readily explain why the increase is overwhelmingly in intrahepatic cholangiocarcinoma or why there are the differences in the age-specific patterns seen in Figure 3.

The reasons behind the increasing incidence of intrahepatic cholangiocarcinoma are unclear. Two recent studies from the USA suggested that the same risk factors could predispose to both the main forms of primary liver cancer (Davila *et al*, 2005; Shaib *et al*, 2005). Shaib *et al* (2005) found hepatitis C infection, alcoholic liver disease and liver cirrhosis, well-established risk factors for hepatocellular cancer, to be strongly associated with intrahepatic cholangiocarcinoma. Intriguingly, using a similar study design, they found comparable associations for the same risk factors and hepatocellular carcinoma (Davila *et al*, 2005). Our findings would, however, suggest that these cancers do not necessarily have similar risk factors.

In conclusion, we have found increases in the incidence of primary liver cancer, which have been particularly dramatic for intrahepatic bile duct cancer, over the last three decades of the 20th century in England and Wales. There has been a halving in the incidence of gallbladder cancer and a reduction of a third in extrahepatic bile duct cancer. Cholangiocarcinoma became the commonest type of primary liver cancer in females in contrast to males, in whom hepatocellular carcinoma remains the most common. The trends in the incidence of intrahepatic bile duct cancer and liver cell cancer follow strikingly different patterns, overall and in the older age groups, suggesting that the two main types of primary liver cancer are unlikely to completely share common aetiologies.

## Figures and Tables

**Table 1:** ICD codes used to identify and group liver, gallbladder and biliary tract cancers

**Definition**	**ICD8 and 9**	**ICD10**	**Abbreviation**
*(a) Subsites*			
Liver, gallbladder and biliary tract	155, 156	C22–C24	TOTAL
Liver cell cancer	155.0	C22.0, C22.2–C22.7	LCC
Intrahepatic bile ducts	155.1	C22.1	IHBD
Liver unspecified	155.2	C22.9	LUS
Gallbladder	156.0	C23	GB
Extrahepatic bile ducts	156.1	C24.0	EHBD
Other parts of the biliary tract	156.2–156.9	C24.1–C24.9	OPBT
			
**Definition**	**ICD-O and ICD-O2**	**Abbreviation**	
*(b) Histological types*			
Cholangiocarcinoma	M816	CCA	
Hepatocellular carcinoma	M817	HCC	
Adenocarcinoma	M814	—	
Carcinoma NOS	M801	—	
Neoplasm NOS+others	M800 and all other codes not specified	—	

ICD, International Classification of Diseases; NOS, not otherwise specified.

**Table 2:** Incidence of cancers of the liver, gallbladder and biliary tract in England and Wales by subsite and sex per 100 000 (using 3-year rolling averages for the calculation of rates)

	**Males**				**Females**			
**Subsite**	**1971–73**	**1999–2001**	**Rate ratio**	**95% CI**	**1971–73**	**1999–2001**	**Rate ratio**	**95% CI**
Liver, gallbladder and biliary tract (total)	3.93	6.13	1.56	1.46–1.66	3.06	3.89	1.27	1.18–1.37
Liver cell cancer	1.84	2.68	1.46	1.31–1.60	0.84	0.91	1.08	0.91–1.26
Intrahepatic bile ducts	0.11	1.33	12.19	10.92–13.46	0.09	1.06	11.94	10.68–13.20
Liver unspecified	0.27^a^	0.58	2.15	1.74–2.56	0.13^a^	0.28	2.07	1.58–2.57
Gallbladder	0.70	0.45	0.65	0.45–0.84	1.19	0.79	0.67	0.54–0.80
Extrahepatic bile ducts	0.86	0.42	0.48	0.32–0.65	0.67	0.36	0.53	0.37–0.69
Other parts of the biliary tract	0.42	0.68	1.61	1.31–1.91	0.27	0.50	1.85	1.50–2.20

CI, confidence interval.

a 1979–1981.

**Table 3:** Subsite by histology: percent of all cases of cancers of the liver, gallbladder and biliary tract in England and Wales within subsite by sex, 2001

	**Males**						**Females**					
**Subsite**	***N* (100%)**	**CCA**	**HCC**	**Adeno**	**Carc**	**NOS+other**	***N* (100%)**	**CCA**	**HCC**	**Adeno**	**Carc**	**NOS+other**
Liver cell cancer	747	0.4	83.7	3.7	2.9	9.2	380	1.3	58.9	9.7	19.7	10.3
Intrahepatic biliary ducts	430	78.8	0.2	2.8	3.5	14.7	455	83.5	0.2	1.5	5.3	9.5
Liver unspecified	143	3.5	9.1	8.4	43.4	35.7	118	5.9	7.6	8.5	38.1	39.8
Gallbladder	146	8.2	0.0	47.3	26.0	18.5	319	4.1	0.3	45.1	33.5	16.9
Extrahepatic bile ducts	108	38.0	0.9	22.2	20.4	18.5	137	30.7	0.7	20.4	24.8	23.4
Other parts of the biliary tract	203	7.4	0.0	57.6	18.2	16.7	197	8.6	0.0	55.8	24.4	11.2

*N*, number of cases; CCA, cholangiocarcinoma; HCC, hepatocellular carcinoma; adeno, adenocarcinoma; carc, carcinoma not otherwise specified; NOS+other, neoplasms not otherwise specified, and all other codes.
